# The evolving view of thermogenic fat and its implications in cancer and metabolic diseases

**DOI:** 10.1038/s41392-022-01178-6

**Published:** 2022-09-16

**Authors:** Xinpeng Yin, Yuan Chen, Rexiati Ruze, Ruiyuan Xu, Jianlu Song, Chengcheng Wang, Qiang Xu

**Affiliations:** Department of General Surgery, State Key Laboratory of Complex Severe and Rare Diseases, Peking Union Medical College Hospital, Chinese Academy of Medical Sciences, Peking Union Medical College, 100023 Beijing, P. R. China

**Keywords:** Cancer metabolism, Cancer microenvironment, Endocrine cancer, Endocrine cancer, Cancer metabolism

## Abstract

The incidence of metabolism-related diseases like obesity and type 2 diabetes mellitus has reached pandemic levels worldwide and increased gradually. Most of them are listed on the table of high-risk factors for malignancy, and metabolic disorders systematically or locally contribute to cancer progression and poor prognosis of patients. Importantly, adipose tissue is fundamental to the occurrence and development of these metabolic disorders. White adipose tissue stores excessive energy, while thermogenic fat including brown and beige adipose tissue dissipates energy to generate heat. In addition to thermogenesis, beige and brown adipocytes also function as dynamic secretory cells and a metabolic sink of nutrients, like glucose, fatty acids, and amino acids. Accordingly, strategies that activate and expand thermogenic adipose tissue offer therapeutic promise to combat overweight, diabetes, and other metabolic disorders through increasing energy expenditure and enhancing glucose tolerance. With a better understanding of its origins and biological functions and the advances in imaging techniques detecting thermogenesis, the roles of thermogenic adipose tissue in tumors have been revealed gradually. On the one hand, enhanced browning of subcutaneous fatty tissue results in weight loss and cancer-associated cachexia. On the other hand, locally activated thermogenic adipocytes in the tumor microenvironment accelerate cancer progression by offering fuel sources and is likely to develop resistance to chemotherapy. Here, we enumerate current knowledge about the significant advances made in the origin and physiological functions of thermogenic fat. In addition, we discuss the multiple roles of thermogenic adipocytes in different tumors. Ultimately, we summarize imaging technologies for identifying thermogenic adipose tissue and pharmacologic agents via modulating thermogenesis in preclinical experiments and clinical trials.

## Background

According to the global epidemiological data from the World Health Organization (WHO), cancer is the first or second leading cause of death in 112 of 185 countries, and it ranks third to fourth and fifth to ninth in an additional 23 and 48 countries, respectively.^1^ Moreover, cancer is also expected to rank as the single most significant obstacle to prolonging life expectancy in the 21st century globally.^2^ Making matters worse, the diagnosis and treatment of cancer were hampered by the worldwide spread of severe acute respiratory syndrome coronavirus 2 (SARS-CoV-2), which resulted in health care setting closures and then possibly lead to a temporary drop in cancer incidence followed by an uptick in advanced-stage disease and consequently increased mortality.^3,4^ For example, cancer screenings for breast and colorectal cancers dropped precipitously at the beginning of the SARS-CoV-2 pandemic, which may affect cancer-related morbidity and mortality.^5^ More sadly, oncologists state that more than 1.9 million new cancer cases and almost 600 thousand cancer deaths are predicted to occur in the United States in 2022 even without taking into account the shock of the SARS-CoV-2 pandemic.^6^ Overall, the burden of cancer incidence and mortality is rapidly growing worldwide.

Cancer cells undergo a reprogramming of metabolism to support biomass production and ATP generation and maintain a redox state in contexts where nutrients of the microenvironment are limiting.^7,8^ To supply pivotal biosynthetic pathways with precursors, the anabolism and catabolism of certain nutrients are upregulated in tumor cells. For example, the Warburg effect was firstly observed in the 1920s, and it occurred in many human tumors.^9,10^ Classical signaling pathways, multiple intracellular and extracellular proteins, plentiful transcription factors, and key metabolic enzymes participate in the regulation of cancer metabolism, and it constitutes a complex interactive network.^11^ Given that, the drug targeted metabolism suggests a potential strategy of precise treatment, and some metabolic drugs currently in clinical trials emphasize the potential effectiveness.^12^ In addition, targeting key regulators involved in metabolic reprogramming can improve response to chemotherapy, radiotherapy, and immunotherapy in various types of cancer.^13,14^

Adipose tissue is generally considered a metabolically active organ with key roles in the modulation of whole-body energy homeostasis, and impairments of its function are directly associated with a variety of metabolic diseases, including obesity, cardiovascular diseases, type 2 diabetes mellitus (T2DM), and cancer.^15,16^ Historically, two types of distinct mature adipocytes—white and brown fat cells were recognized to exist in humans. Because of the difference in morphology and function, white adipocytes are major energy storage sites, while brown adipocytes function as burning energy.^17^ Significantly, another type of adipocytes appears in white fat depots but these fat cells exhibit a substantial capacity for induction of thermogenesis. Given that these adipocytes and classical brown fat derive from different cellular lineages and thus, this specific type of adipocytes is called beige adipocytes, and the process of white-to-beige conversion is called the browning of white adipose tissue (WAT).^18,19^ Current evidence showed that altered thermogenic fat consisting of brown and beige adipocytes was related to metabolic diseases, such as overweight and T2DM, and targeting thermogenic mechanisms can offer a new therapeutic strategy.^20,21^

The mask of thermogenic fat abnormality in cancer is being gradually unveiled due to a better understanding made advances in the cellular and functional complexity of thermogenic adipose tissue. However, how thermogenic adipose tissue affects the biological behavior of cancer cells remains obscure. So in this review, we concentrate on clarifying the origin and physiological functions of thermogenic fat as well as its role in tumors and emphasizing the potential clinical application value of thermogenic adipose tissue.

## The developmental origin and anatomical location of thermogenic adipocytes

**Fig. 1:**
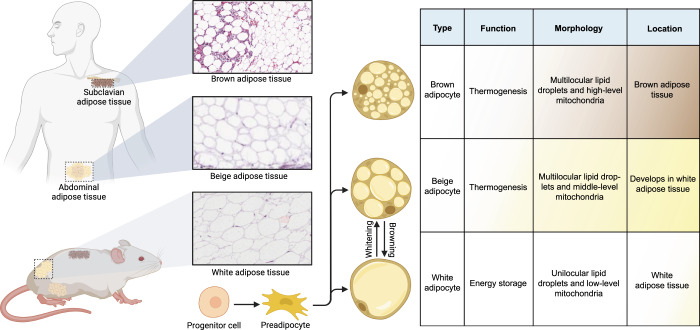
The biogenesis of different adipocytes and their characteristics. The three mature adipocytes including white adipocytes, beige adipocytes, and brown adipocytes can derive from progenitors via de novo differentiation. Importantly, white adipocytes can reinstall the thermogenic program by mitochondria biogenesis in response to cold and certain other stimuli. When external stimuli are withdrawn, mitochondria-enriched beige adipocytes transform into dormant adipocytes that resemble white adipocytes. In addition, the three types of adipocytes have distinct morphology and anatomical location. White adipocytes are mostly distributed in white adipose tissue, existing in various subcutaneous and intra-abdominal depots and contributing to the storage and release of energy. Stimulated by beigeing factors, beige adipocytes appear in white adipose tissue sporadically. Compared with beige adipocytes, brown adipocytes are an embryonic-origin cell type and cluster in designated depots like interscapular brown adipose tissue depots of mice and infants. However, both brown and beige fat cells are capable of thermogenesis because they have multilocular lipid droplets and densely packed mitochondria. Part of the HE images in this figure is generated from the Human Protein Atlas. This figure was created on BioRender.com with permission for publication

Generally, the defining feature of active thermogenic adipocytes has been the expression of the mitochondrial protein uncoupling protein 1 (UCP1) and multilocular lipid droplet appearance. Therefore, both brown and beige adipocytes belong to thermogenic fat cells. Despite these similarities, beige and brown adipocytes are recognized as two distinct cell types because each of them has unique biological characteristics beyond thermogenesis, suggesting cellular heterogeneity of thermogenic fat^22–24^ (Fig. 1).

### Origin of brown adipocytes

In mammals, the developmental origin of classical brown fat cells is evolutionally conserved. Concretely, brown adipocytes develop prenatally, and their fate is determined by mid-gestation. It implies that the thermogenic function of brown adipose tissue (BAT) is completely activated at birth, which is of supreme importance to newborn animals because neonates of many larger animals including humans are based on the non-shivering thermogenesis of BAT to maintain normal body temperature.^25–27^ Pulse-chase lineage tracing studies suggest that most brown fat cells originate from precursor cells in the embryonic mesoderm developmentally, and these precursors transiently express somite markers, such as paired-box protein 3 (Pax3), myogenic factor 5 (Myf5), and mesenchyme homeobox 1 (Meox1), engrailed 1 (En1).^17,28^ In rodents, the major BAT depots are distributed in the interscapular region covering axillary, interscapular, and cervical pads embedded in and around deep back muscles. Some of the BAT depots in humans are anatomically analogous to those in rodents. In addition to the three pads mentioned above, humans also possess BAT depots in four anatomic regions, such as abdominal, mediastinal, supraclavicular, and paraspinal. However, some of the BAT depots regress and are absent in adults, for example, the BAT depot in the interscapular region is most dominant in infants and gradually declines with growing.^29–31^

### Origin of beige adipocytes

Although beige adipocytes share many morphological and biochemical characteristics with classical brown adipocytes, they have distinguishing phenotypic and functional features.^32^ Compared to brown adipocytes, beige adipocytes develop postnatally, and they are a recruitable cell type derived from non-dermomyotome cells in WAT depots.^33^ It is currently accepted that the recruitment of beige adipocytes occurs in two ways. Primarily, beige fat cells can arise via de novo differentiation from adipocyte progenitors. Then, the rouse of the thermogenic phenotype by dormant cells also contributed to the recruitment of beige adipocytes in WAT depots.^15^ It is commonly recognized that both direct de novo biogenesis and white-to-beige conversion engaging progenitor cells occur in vivo, with the contribution from each pathway influenced by experimental conditions. For example, the researchers used the model of Adipo-Chaser (inducible adiponectin–Cre lineage tracing) mice and suggested that the majority of beige adipocytes arose from de novo biogenesis when the mice are at thermoneutrality (30 °C) and subsequently exposed to cold (6 °C). By contrast, if the mice are transferred from ambient temperature (20–23 °C) to cold (6 °C), only almost half of the beige fat cells are derived via de novo adipogenesis, with the remaining beige adipocytes generating from the dormant adipocytes.^23,34,35^ Except for the influence of external cues, the mechanism of recruiting beige adipocytes may vary depending on the heterogeneity of progenitors. As an illustrative example, cold, as the most well-known thermogenic stimulus, can promote de novo beige adipocyte differentiation from α smooth muscle actin (αSMA)-positive stromal progenitor cells through triggering intracellular signaling, including cyclic adenosine monophosphate (cAMP) signaling via stimulation of β-adrenergic receptor (β-AR). By contrast, myogenic PDGFRα + progenitors can be activated by thermal stress in the absence of β-AR signaling and turn to beige fat.^36–38^ In addition to the existence of multiple types of progenitors, beige adipocytes are composed of several subpopulations, such as glycolytic beige fat using mostly glucose as a metabolic fuel, and lipolytic beige fat tending to consume free fatty acids (FAs).^39,40^ Intriguingly, there is an important phenomenon: the recruited beige fat cells can transform into dormant adipocytes with white fat characteristics morphologically and functionally when the external stimulus is removed, and this process is referred to as beige-to-white conversion.^41,42^ In terms of the distribution of beige fat throughout the body, beige adipocytes sporadically reside within WAT depots in the postnatal stage. It is universally accepted that beige adipocytes exist mainly in subcutaneous WAT, including anterior subcutaneous and inguinal WAT, and suprascapular fat depots.^17,26,43^ However, the developmental features of thermogenic fat cells in some anatomical sites remain obscure. For example, perivascular adipose tissue (PVAT) was widely perceived to be brown fat depots. Nevertheless, recent studies suggest that PVAT shows characteristics of beige adipose tissue in humans and BAT-like in mice. More precisely, PVAT is not always brown-like in mice or beige-like in humans, depending on the anatomic location and environmental or metabolic context.^44,45^

In sum, the reinstallation and loss of thermogenesis in beige fat are adapted to altered external conditions. The mechanism of recruiting beige adipocytes may vary depending on the nature of the stimulus and of heterogeneity beige adipocytes in various metabolic diseases including cancer, which will be infusive areas for future research.

### The multifaced roles of thermogenic fat

**Fig. 2:**
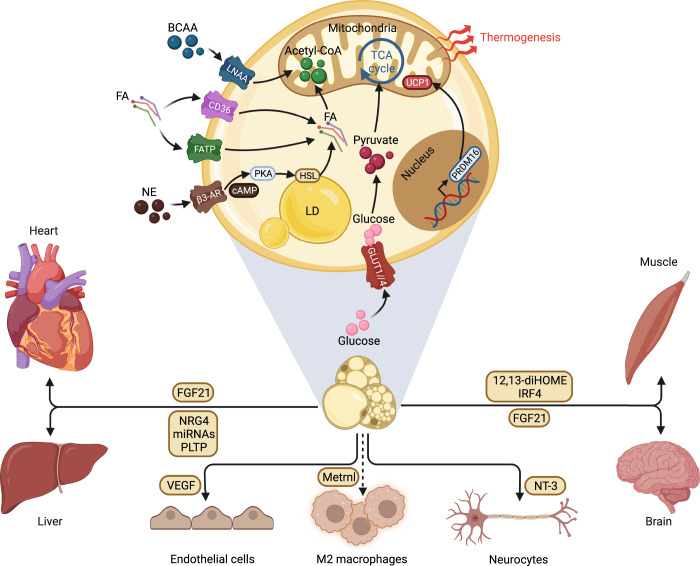
The multiple roles of thermogenic adipocytes in metabolic homeostasis. Thermogenic fat is generally considered a metabolic sink for glucose, lipid, and BCAAs. In addition, brown or beige adipocytes have an impact on neighbor cells by secreting batokines. Moreover, thermogenic adipose tissue is also recognized as an endocrine organ, regulating the gene expression or functions in distant organs, such as the heart, liver, muscle, and brain. BCAA branched-chain amino acids, LNAA the large neutral amino acid transporter, FA fatty acid FATP fatty acid transport protein, NE norepinephrine, β3-AR β3-adrenergic receptor, PKA protein kinase A, HSL hormone-sensitive lipase, LD lipid droplet, TCA tricarboxylic acid, UCP1 uncoupling protein 1, PRDM16 PR domain containing 16, GLUT1/4 glucose transporter 1/4, FGF21 fibroblast growth factor-21, NRG4 neuregulin 4, PLTP phospholipid transfer protein, VEGF vascular endothelial growth factor, Metrnl meteorin-like hormone, NT-3 neurotrophin 3. This figure was created on BioRender.com with permission for publication

The most important biological role of brown and beige fat cells is to participate in the process of non-shivering thermogenesis that involves uncoupling protein 1 (UCP1, a characterized thermogenic factor highly expressed in beige and brown adipocytes)-dependent and UCP1-independent mechanisms.^46^ Beyond the capability of producing heat, thermogenic fat is also a metabolic pool for glucose, lipid, and branched-chain amino acids (BCAAs).^47^ In addition, the accumulation of brown or beige adipose tissue is coupled with anti-inflammation, anti-fibrosis, and angiogenesis. Moreover, thermogenic fat considered a secretory organism can secrete various molecules to mediate communication with diverse organs and tissues via autocrine, paracrine, and endocrine.^48,49^ Numerous preclinical studies and comprehensive reviews on the biofunction of thermogenic adipose tissue in physiology have been reported and will be discussed briefly here. Instead, we focus on the influence caused by breaking brown and beige fat homeostasis on metabolic diseases (Fig. 2).

### Non-shivering thermogenesis

The mechanisms of thermogenesis consist of classical UCP1-dependent and novel UCP1-independent pathways.^50^ As the name implies, the activation of the former pathway is mediated by UCP1 and relies on the transcription co-regulatory protein PR domain containing 16 (PRDM16).^51^ The futile metabolic cycling mechanisms consist of Ca^2+^ cycling, creatine-dependent substrate cycling, and triacylglycerol (TAG) futile cycle are the basis of UCP1-independent thermogenesis, which has no other effect but the consumption of ATP and dissipation of energy.^52–55^

Intimately closed to the malfunction of thermogenesis, obesity is often described as a disorder of energy intake and expenditure.^56^ Up to now, abundant evidence has proved the fact that impaired heat production can give rise to obesity. For example, inhibition of the key transcriptional factor PRDM16 or reduction of UCP1 which is both involved in the UCP1-dependent pathway can drive obesity.^57,58^ Similarly, in the process of UCP1-independent thermogenesis, suppression of any one of the three futile metabolic cycling mechanisms can cause obesity. For instance, genetic depletion of creatine metabolism in adipocytes impairs diet-induced thermogenesis that limits weight gain in response to caloric excess and then develops obesity.^59^ Importantly, obesity is a high-risk factor for metabolic diseases, including angiocardiopathy, T2DM, and malignancy.^60–62^ Therefore, induction of the browning process to enhance the capability of thermogenesis in the whole body holds a promising therapeutic potential to combat obesity and its complications.^48,63,64^

### Roles in glucose metabolism

As an important substrate for fueling thermogenesis, glucose can be actively transported into thermogenic adipocytes which is a common characteristic. Typically, glucose uptake in beige and brown adipocytes is stimulated by insulin. In this process, thermogenic adipocytes serve as a cellular ‘rheostat’ that senses the glucose status and contributes significantly to whole-body energy homeostasis.^65,66^ Given that brown and beige adipocytes can take up glucose positively, ^18^F-fluorodeoxyglucose positron emission tomography combined with computed tomography (FDG-PET/CT) was used as an effective method to characterize and quantify thermogenic depots volume in humans, and the prevalence of thermogenic adipose tissue assessed by ^18^F-FDG-PET/CT was highly correlated with a lower incidence of T2DM.^60,67–69^

Insulin stimulation of glucose uptake via glucose transporters in fat tissues is essential for regulating systemic blood glucose levels. Concretely, glucose transporter type 4 (GLUT4) known as the predominant insulin-responsive glucose transporter redistributes within adipocytes in response to insulin, and this net redistribution of GLUT4 from intracellular storage to the plasma membrane is generically called “GLUT4 translocation”.^70–72^ For example, defective GLUT4 translocation or inhibition of GLUT4 in adipocytes induces systemic glucose homeostasis dysregulation and leads to a risk of developing T2DM.^73,74^ Importantly, thermogenic fat cells can take in more glucose via the GLUT1 than that white fat cells solely rely on GLUT4.^17,75^ So, given the significant role of thermogenic adipocytes performed in regulating glucose, the impaired capability of thermogenic fat tissue may give rise to metabolic disorders in vivo. Indeed, evidence from an early study demonstrated that genetic deletion of brown adipocytes leads to the development of obesity.^76^ Besides, researchers found that brown fat transplantation significantly reduces fat mass and enhanced glucose metabolism and insulin sensitivity in mice models.^43,77^ Similarly, it has been proved that elevated UCP1 levels in human white adipocytes improve glucose uptake by 40%.^78^ Moreover, a recent study found that PRDM16, the key transcriptional regulator of beige adipocytes de novo synthesis, in SAT was significantly lower in T2DM and prediabetes as compared to the normal glucose tolerance group.^79^ Consistently, PRDM16 transgenic mice showed enhanced glucose tolerance, high energy expenditure, and limited weight gain in response to high-fat feed.^80^ Therefore, a wealth of evidence mentioned above suggests that targeting brown or beige fat cells may have therapeutic utility in disorders of glucose homeostasis. For example, treatment with the β3-adrenergic receptor (β3-AR) agonist, such as mirabegron which was approved for treating overactive bladder, can improve glucose and insulin homeostasis.^81–83^

### Roles in lipid metabolism

Although thermogenic fat is characterized by a high rate of glucose uptake, FAs are generally recognized as the primary fuel for mitochondrial uncoupling respiration. In general, upon activation by long-chain FAs, UCP1 increases the conductance of the inner mitochondrial membrane (IMM) to make thermogenic adipocytes mitochondria generate heat.^30,84,85^ As for the source of FAs, it is believed that it is mainly released from WAT rather than de novo lipolysis in BAT which was initially thought to be the primary fuel source.^86,87^ The BAT takes up FAs via plasma membrane FA transport proteins such as CD36 or FAs transport protein 1 (FATP1), and impairment of FATP1 inhibits FA uptake into BAT and suppresses thermogenesis. By contrast, promoting the translocation of the FA transporters FATP1 and CD36 to the cell membrane can increase FAs uptake into thermogenic adipocytes.^88,89^ Therefore, ^18^F-fluoro-thiaheptadecanoic acid (^18^F-THA)-PET/CT is applied to assess the activity of brown or beige fat tissues.^90^

Given the fact that the FAs constitute a preferred energetic substrate for most thermogenic adipocytes, increasing evidence has shown that thermogenic fat exerts effects on systemic lipid metabolism. As illustrative examples, BAT activity can control vascular lipoprotein homeostasis by boosting triglyceride-rich lipoproteins (TRL) turnover and channeling lipids into BAT, and this process crucially relies on local lipoprotein lipase (LPL) activity and CD36 in mice models by short-term cold exposure.^91^ In line with this, BAT after both acute cold and a highly selective β3-AR agonist CL316,243 treatment accelerates intravascular LPL-mediated lipolysis of TRL, and TRL-derived FAs are the most prominent energy source for energy replenishment in BAT.^92^ More recently, researchers found that endothelial cells of BAT internalize entire TRL particles and follow the endosomal pathway for lysosomal acid lipase (LAL)-dependent hydrolysis.^93^ Further, impaired thermogenesis of mice showed a remarkable increase in plasma triglyceride levels in response to cold exposure.^94^ In addition to decreasing pro-atherogenic remnant lipoproteins in hyperlipidemic mice, both cold-induced and pharmacological thermogenic activation confers atheroprotective properties via increasing the levels of high-density lipoprotein (HDL) cholesterol and promoting reverse cholesterol transport.^95^ Besides, a more recent clinical trial study comes to a similar conclusion that human BAT metabolic activity can be improved after mirabegron treatment chronically and consequently contribute to elevations of the beneficial lipoprotein biomarkers in plasma like HDL and apolipoprotein A1 (ApoA1), as well as total bile acids.^96^ According to current evidence, activation of brown or beige adipose tissue seems to be beneficial to human health through regulating lipid metabolism. Of note, current research also emphasizes the importance of UCP1-independent thermogenesis based on ATP-dependent futile cycling, and how it impacts the whole-body lipid homeostasis dynamically remains unclear.

### Roles in amino acid metabolism

Apart from glucose and FAs, BCAAs including valine, leucine, and isoleucine have also been demonstrated to support thermogenesis in mice and humans, and active BCAAs oxidation is required for optimal thermogenesis in turn.^97^ As an illustrative example, BAT positively uptake and catabolize BCAAs to produce heat in febrile mice and rats.^98^ Additionally, BAT actively promotes BCAAs clearance in circulation via utilizing BCAAs in the mitochondria for thermogenesis in mice and humans in response to cold exposure. Moreover, defective BCAAs catabolism in beige and brown adipose tissue causes impaired thermogenesis.^97^ In line with this, knocking out genes associated with heat production in mice displays an inability to thermoregulate and aberrant BCAAs and FAs metabolism.^99^ Sadly, several previous studies have suggested that high BCAAs levels in plasm and impaired metabolism of BCAAs are associated with the occurrence of T2DM,^100–102^ partially related to activating the mammalian target of rapamycin complex 1 (mTORC1) and protein kinase Cε (PKCε).^103^ Remarkably, BCAAs possibly have a significant impact on adipocyte differentiation more than a simple energetic fuel in thermogenic adipocytes.^104^ Compared to proliferating cells, differentiated adipocyte cells displayed enhanced BCAAs catabolism, and inhibition of BCAAs catabolism compromised adipogenesis.^105^ More precisely, Sirtuin 4 (SIRT4) enhances BCAAs catabolism by activating methylcrotonyl-coenzyme A carboxylase (MCCC), then increased BCAAs catabolism stimulates peroxisome proliferator-activated receptor-gamma (PPARγ) that is a pivotal regulator of adipogenesis. Further, decreased SIRT4 expression is found in the adipose tissue of diabetic mouse models.^106^ To sum up, although an interactive network was initially established among thermogenic fat, BCAAs, and metabolic diseases, the underlying mechanisms remain obscure. So to comprehensively understand the roles they play calls for further experiments and clinical trials.

### Secretory functions act locally and distantly

Beyond heat generation, solid evidence has suggested that some of the physiological effects of beige and brown adipocytes are mediated by releasing small molecules, defined as batokines. Some of the adipokines released by brown or beige adipocytes have been identified by the transcriptomic, proteomic, and metabolomic analysis, including proteins, lipids, and metabolites. Furthermore, the broad number of articles suggest that these secretory batokines mainly act in an autocrine, paracrine, or endocrine manner to regulate neighboring cells and distant organs.^107–111^

Generally, batokines can act on cells within the adipose tissue and promote adipogenesis, angiogenesis, neurite outgrowth, and immune cell interactions. In particular, the thermogenic activity of beige and brown fat cells can be enhanced by bone morphogenetic protein-8b (BMP8b), fibroblast growth factor-21 (FGF21), Follistatin-like 1 (FSTL1), and the cytokine interleukin-6 (IL-6), or inhibited by the soluble form of the LDL receptor 11 (sLR11), which are secreted by themselves with autocrine actions.^112–116^ Additionally, other small molecules secreted by brown or beige adipocytes regulate locally other cell types.^117^ For example, neurotrophin 3 (NT-3) and vascular endothelial growth factor (VEGF) secreted by thermogenic adipocytes can promote sympathetic innervation and target endothelial cells to induce vascularization of brown and beige adipose tissue, respectively.^118–120^ Besides, thermogenic adipocytes also have an impact on immune cells through releasing meteorin-like hormone (Metrnl) which promotes eosinophil activation to produce IL-4, leading to the recruitment of alternatively activated M2 macrophages and the increased expression of anti-inflammatory gene programs.^121–123^

Beyond these local effects, batokines can impact distant tissues in an endocrine fashion, such as the liver, heart, skeletal muscle, and central nervous system (CNS).^110^ Recently, plenty of studies have shown that thermogenic adipocytes can target CNS and subsequently regulate systemic energy balance and food intake. Furthermore, batokines such as FGF21 might influence sympathetic nervous system activity and circadian behavior.^124,125^ As an interactive network, CNS also has an influence on the browning of adipose tissue and facilitating heat generation. As an illustrative example, researchers newly have demonstrated that a population of GABAergic neurons in the dorsolateral portion of the dorsal raphe nucleus (DRN) is capable of regulating thermogenesis through both direct and indirect pathways.^126,127^ Given current evidence, the browning of fat is beneficial to cardiometabolic. Preclinical experiments have suggested that some of the factors released by thermogenic fat cells such as FGF21 increase cardiac substrate oxidation and protect the heart from hypertensive cardiac remodeling.^128^ Consistent with this result, a large retrospective study published lately demonstrated that the presence of thermogenic adipocytes is correlated to a low prevalence of cardiovascular diseases, including hypertension, coronary artery disease, congestive heart failure, and cerebrovascular disease.^60^ Current evidence also suggests the crosstalk between thermogenic adipocytes and hepatocytes through batokines. More precisely, activated thermogenic adipocytes secret neuregulin 4 (NRG4), which then acts on hepatocytes to decrease de novo lipogenesis and protect the liver from damage.^129^ Alcohol consumption or direct alcohol administration can stimulate BAT to secrete some adipokines, such as adiponectin, which suppresses hepatocyte injury and death.^130^ Furthermore, a recent study showed that BAT is a major source of exosomal miRNAs in humans and mice, and several microRNAs packaged into extracellular vesicles such as microRNA (miR)-99b may inhibit hepatic FGF21 generation.^131^ Besides, researchers identified a previously ignored batokine phospholipid transfer protein (PLTP) by using proteomics and transcriptomics in human thermogenic adipose tissue, which regulates metabolism in the liver.^132^ In addition, there is some documentation of crosstalk between thermogenic adipose tissues and skeletal muscle in mammals. For instance, the FA derivative 12,13-dihydroxy-(9Z)-octadecenoic acid (12,13-diHOME) is increased within beige and brown fat in response to exercise or cold exposure to enhance thermogenesis, leading to the enhanced FAs uptake and oxidation of myocytes in an endocrine manner.^88,133^ Moreover, loss of interferon regulatory factor 4 (IRF4) induces myogenic gene expression like the secreted factor myostatin in BAT, a classical muscle mass negative regulator that results in impaired mitochondrial function and diminished exercise capacity.^134^

Collectively, these publications have identified plenty of candidate batokines from mammals, and some of them remain mysterious. Further research is warranted into this aspect to comprehensively understand the secretome of beige or brown adipocytes, and describe the action mode of each of these molecules in every single metabolic disease, including malignancy which is seem to be forgotten by researchers studying thermogenic fat.

### The regulation of thermogenic fat in metabolic diseases

Currently, it is widely accepted that the activity of thermogenic adipose tissue declines during the development of metabolic disorders.^29,135^ Therefore, thermogenic fat is emerging as an attractive and promising target for therapeutic intervention in metabolic diseases like obesity and T2DM, because of its capacity to utilize glucose and lipids for thermogenesis and release molecules locally or distantly that contribute to a hypermetabolism state of the whole body.

### Obesity

Obesity is defined by the WHO as excessive fat accumulation that might impair health and is diagnosed at a body mass index (BMI) ≥30 kg/m^2^.^136^ The prevalence of obesity has already reached pandemic levels worldwide, thereby substantially increasing the risk of other metabolic diseases such as T2DM, fatty liver disease, hypertension, and several cancers.^137^ These non-communicable diseases(NCDs) lead to a lower standard of living and a decline in life expectancy.^138^ In terms of pathogenesis, the fundamental cause of obesity is a long-term energy imbalance between too many calories intaked and too few calories expended. Examples, overeating (like emotional eating, peer pressure, snacking), low energy expenditure (like aging, neuroendocrine factors, sarcopenia, and medications), or physical inactivity (like chronic fatigue, low fitness level, emotional barriers, and joint pain) can influence the chronic positive energy balance, thus subsequently causing obesity.^139,140^ Besides, obesity might be considered a heritable trait. The heritability of BMI has been estimated as 30–40% based on a recent study.^141^ Furthermore, some investigations found that loss of function in genetic levels for leptin, leptin receptor, melanocortin 4 receptor, and others might cause severe obesity in humans.^142,143^ Therefore, obesity most likely results from a concerted interplay of genetic and environmental factors.

During the development of obesity, adipose tissue can expand by de novo synthesis and enlargement of existing adipocytes, whereas the mass of activated thermogenic fat is decreased. Compared with white fat, brown and beige adipose tissue is beneficial for combating obesity by enhanced lipolysis of triglycerides and the oxidation of FAs.^144,145^ Previous research discovered that expression of thermogenic genes reduced and adipocytes shifted from multilocular to unilocular appearance in mice fed a high-fat diet compared to mice fed a normal diet.^146^ Consistently, investigators found that lean men had more activated BAT than obese men by using ^18^F-FDG-PET/CT.^29^ The reason for the reduced thermogenesis in obesity is that B lymphocytes and macrophages infiltrate thermogenic fat, and subsequently suppress UCP1 expression via releasing tumor necrosis factor-α (TNFα).^147–149^ In turn, impaired thermogenesis of adipose tissue can drive obesity. Give an illustrative example, selectively genetic depletion of glycine amidinotransferase (GATM) in fat (Adipo-Gatm KO) or the cell surface creatine transporter (CRT) in fat (AdCrTKO) substantially reduces adipocyte creatine, inhibits thermogenesis and energy expenditure, then driving obesity as a consequence.^59,150^ Consistently, adipocyte-selective inactivation of creatine kinase B (CKB, is indispensable for futile creatine cycling-based thermogenesis) in mice disturbs glucose homeostasis and diminishes thermogenic capacity, subsequently increasing predisposition to obesity.^55^ Therefore, stimulating the thermogenesis of adipose tissue is an effective approach with which to curb obesity.^151^ As a classical external clue, cold can upregulate thermogenic gene expression of human fat via inducing the secretory of irisin and FGF21.^152^ Besides, the cold-induced 12,13-diHOME increases FAs uptake into brown adipocytes by promoting the translocation of the FA transporters FATP1 and CD36 to the cell membrane, which resulted in decreased levels of serum triglycerides.^88^ In addition to cold, exercise also can induce WAT lipolysis and BAT thermogenesis in vivo by increasing the plasma level of the tricarboxylic acid cycle intermediate α-ketoglutaric acid (AKG) which is also negatively correlated with BMI. Mechanically, the AKG stimulates the secretion of adrenaline through 2-oxoglutarate receptor 1 (OXGR1) expressed in adrenal glands and causes muscle hypertrophy and fat loss consequently.^153^ Moreover, refeeding-induced mesencephalic astrocyte-derived neurotrophic factor (Manf) curbs diet-induced obesity by directly promoting the browning of adipocytes via the p38 MAPK pathway.^154^ Of note, a variety of phytochemicals have been shown in the literature to counteract weight gain via adipose thermogenesis.^155^ Currently, investigators identified phytochemical hyperforin (HPF) as an agent to combat obesity via using the Connectivity Map (CMAP) approach. More specifically, they found that HPF directly targeted dihydrolipoamide S-acetyltransferase (DLAT) and thereby enhanced the capacity of heat generation by activating AMPK and PGC1A.^156^ However, stimulating the thermogenesis of the whole body has limited applications because of the potential risk of cardiovascular diseases and other complications caused by hypermetabolism.^157,158^ Thus, safer approaches to achieving beige fat activation are required. Polyethylene glycol (PEG)-crosslinked polydopamine nanoparticle (PDA) is a safe and injectable photothermal hydrogel that converts near-infrared (NIR) light input into accurately controlled temperature output.^159,160^ Recently, researchers achieved local hyperthermia of fat in vivo by using PEG-PDA hydrogel and treated obesity without adverse effects as a consequence. Mechanically, local hyperthermia activates heat shock factor 1 (HSF1), and enhanced HSF1 regulates *Hnrnpa2b1* transcription, consequently increasing the mRNA stability of key metabolic genes.^161^ In conclusion, the manipulation of thermogenic activity in adipose tissue is regarded as a potential strategy in the treatment of obesity.

### T2DM

T2DM is arguably one of the largest epidemics ever seen globally, leading to the ninth major cause of death. The number of people with T2DM has doubled in the last several decades and is projected to rise further to almost 700 million by 2045.^162,163^ Worryingly, the incidence and prevalence of T2DM in younger adults (aged <40 years) have risen sharply since the 2000s.^164^ Young-onset T2DM presents a more aggressive disease phenotype because it has a more severe and rapid deterioration of β-cell function and is more likely to develop complications.^165^ The reasons for the escalating epidemic of T2DM are multiple, including population aging, economic development, a sugar-rich diet, and a sedentary existence.^166^ For example, the incidence of T2DM increases proportionally with BMI.^167^ Besides, large prospective studies have shown that the rise in body weight over time dramatically fuels the prevalence of T2DM.^168^ Consistently, BMI exceeding the upper limit (25 kg/m^2^) was associated strongly and positively with mortality attributed to diabetes.^136^ In conclusion, substantial evidence directly points out that adiposity has an impact on the development of T2DM.^169^

Accumulation of excess WAT is detrimental to metabolic health, while the activation of thermogenic fat has a beneficial influence on diabetes.^170^ Indeed, plenty of research has demonstrated that glucose uptake is increased in activated thermogenic fat.^67,135^ Moreover, cold-induced activation of thermal adipose tissue improves overall glucose disposal and insulin sensitivity.^171–173^ Give an illustrative example, overweight men with T2DM who received a short-term cold acclimation appeared to enhanced thermogenic activity and improved whole-body insulin sensitivity.^172^ In general, thermogenic adipose tissue intakes glucose from circulation, contributing to glucose clearance and the decreased demand for insulin secretion by β-cells.^174^ Mechanically, boosting glucose uptake into brown or beige adipose tissue consists of two different mechanisms: insulin-dependent and insulin-independent. Insulin is a major regulator of glucose uptake in most tissues, including BAT, WAT, and skeletal muscle glucose. However, heat production-related glucose uptake into thermogenic fat has been suggested to be independent of insulin signaling, primarily dependent on GLUT1 transporter in an adrenergic-promoted manner.^175–177^ In addition to decreasing glucose directly, peripheral lipid clearance also indirectly benefits β-cells and aids in restoring peripheral insulin sensitivity.^178^ Collectively, beige and brown fat confers beneficial effects on glucose metabolism and insulin sensitivity, which may be used as an underlying therapeutic target for the treatment of T2DM.

### Cardiovascular diseases

Non-communicable diseases (NCDs) are the leading cause of death and ill health, which account for seven of ten deaths around the world.^179^ Of the NCDs, cardiovascular disease (CVD) is now the leading cause of mortality and morbidity worldwide.^180^ In China, CVD causes 40% of deaths in the Chinese population.^181^ Over 95% of all CVD deaths are attributable to six conditions: ischemic heart disease (IHD), stroke, heart failure, cardiomyopathy, rheumatic heart disease (RHD), and atrial fibrillation (AF).^182,183^ Of note, the incidence of CVD has increased among young adults (defined in general as individuals aged 18–45 years) in the past 2 decades, largely because of a high prevalence of risk factors for CVD, such as obesity, physical inactivity, and poor diet.^184^ Indeed, obesity contributes directly to the incidence of cardiovascular risk factors like dyslipidemia, T2DM, and hypertension. Further, obesity also leads to CVD mortality independently of other cardiovascular risk factors.^185^ Based on epidemical data, the prevalence of obesity was responsible for 4 million deaths in 2015, with two-thirds of this number attributed to CVD.^186^ Specifically, CVD caused by obesity is closely related to excess adipose accumulation and metabolic abnormalities. As an active endocrine and paracrine organ, excessive adipose tissue releases multiple hormonses and cytokines, such as leptin, adiponectin, IL-6, and TNF-α, which result in diabetes, cardiovascular inflammation, increased blood pressure level, fibrinolysis, and atherosclerosis.^187–189^ Accordingly, physical activity can attenuate the adverse effects of obesity on CVD events.^190^

Generally, thermogenic fat potentially exerts beneficial metabolic and cardiovascular effects through stimulating energy expenditure, attenuating cardiac remodeling, and suppressing the inflammatory response.^191–193^ Firstly, the activation of thermal fat plays a protective role in the vascular system. In animal studies, increased BAT activation stimulated by beta-adrenaline can diminish the progress of hypercholesterolemia and protect from atherosclerosis development in mice with hyperlipidemia.^39^ Consistently, researchers used ^18^F-FDG-PET/CT to evaluate the relationship between ^18^F-FDG uptake in supraclavicular BAT to arterial inflammation and subsequent CVD events in humans, and the results suggested that increased supraclavicular BAT activity is inversely associated with arterial inflammation.^194^ In addition, PGC1A can not only assist carbon monoxide to complete vasodilation but also regulate vascular senescence negatively.^195,196^ Remarkably, most vessels in the body are surrounded by PVAT which shares thermogenic adipose-like properties like high mitochondrial density and increased expression of UCP1.^197^ In a previous study, investigators found that activation of PVAT during cold acclimation improved endothelial dysfunction and attenuated the development of atherosclerosis.^198^ In line with it, knockdown of BMP4 (which transforms white adipocyte to beige adipocyte) in PVAT inhibited the expression of thermogenic genes and aggravated endothelial inflammation in a co-culture system. Accordingly, overexpression of *BMP4* in adipose tissues enhanced the thermogenic activity of PVAT and protects against atherosclerosis in mice.^199^ Moreover, the PVAT also secretes multiple vasorelaxant factors such as adiponectin,^200^ gasotransmitters hydrogen sulfide,^201^ nitric oxide,^202^ and palmitic methyl ester,^203^ and these vasorelaxant factors can alleviate blood pressure in the microcirculatory system. Of note, epicardial adipose tissue (EAT) is part of the VAT that surrounds the heart, excessive accumulation of whom is considered a risk factor for the incidence of coronary artery diseases.^204–206^ Currently, multiple reports of UCP1, PGC1A, and PRDM16 expression have established their presence in human EAT, which functionally corresponded with downregulation in the production of reactive oxygen species and immune responses. Furthermore, thermogenic genes expressions in EAT were recongnized as protective factors against coronary artery disease and heart failure with reduced left ventricular ejection fraction.^207–209^ Secondly, brown or beige adipose tissue are capable of improving cardiac remodeling. By contrast, mice with genetic ablation of UCP1 is susceptible to hypertension, cardiomyopathy, and fibrosis.^210^ In line with it, compared with the control group, UCP1 knock-out mice displayed fibrosis, augmented myocardial injury, and decreased survival rates when isoproterenol was administered. Interestingly, the cardiac parameters and survival of UCP1 knock-out mice were improved after receiving a BAT transplant from the controls.^211^ The molecular mechanism underlying the role of BAT in pathological cardiac remodeling is mediated by FGF21. Specifically, activation of the adenosine 2 A (A2A) receptor in thermogenic adipocytes can attenuate hypertensive cardiac remolding by inducing themselves to release FGF21.^128,212^ Incidentally, factors like natriuretic peptides produced by the heart could regulate the thermogenic activity of adipocytes via the cyclic guanosine monophosphate (cGMP)–protein kinase G (PKG)–p38 signaling pathway.^213^ Finally, thermogenic fat can cause a decline in the incidence of CVD and associated adverse events by reducing glucose and lipid levels.^214^ According to the characteristics of thermogenic fat, it can be speculated that stimulating the browning of adipose tissue in a specific region can be protective for cardiovascular system.

### The crosstalk between thermogenic adipose tissue and cancer

**Fig. 3:**
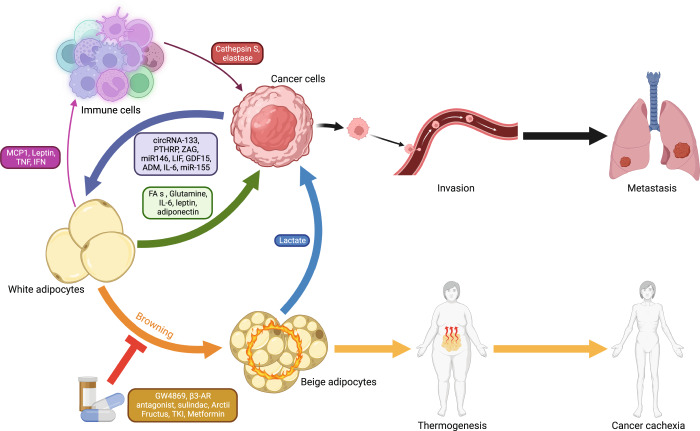
The role of thermogenic adipose tissue in malignancy. Polypeptides, metabolites, or other certain mediators derived from cancerous cells initial the conversion of white-to-beige locally and distantly. On the one hand, activated beige adipocytes in SAT lead to lipolysis and energy expenditure, subsequently contributing to cancer-associated cachexia. On the other hand, the adjacent thermogenic fat can directly promote tumor growth and metastasis by secreting specific molecules, such as lactate. Accordingly, pharmacologically inhibiting the browning of white adipose tissue slows cancer progression and improves the outcomes for patients. ZAG zinc-α_2_-glycoprotein, PTHRP parathyroid-hormone-related protein, LIF leukemia inhibitory factor, GDF15 growth differentiation factor 15, ADM adrenomedullin, IL-6 the cytokine interleukin-6, β3-AR β3-adrenergic receptor, TKI tyrosine kinase inhibitor, Arctii Fructus the extract of Arctium lappa. This figure was created on BioRender.com with permission for publication

There is growing evidence to indicate that the dysregulation of adipose tissue is closely linked with metabolic diseases in rodents and humans, such as T2DM, obesity, fatty liver, and pancreatitis. Besides, some of them have been considered high-risk factors for multiple tumors. Recently, there is a systematic understanding that WAT is capable of promoting tumor growth, metastasis, and chemoresistance.^16,215–217^ In short, WAT generally accelerates tumor progression through the following three pathways. Firstly, white adipocytes fuel tumor growth by providing nutrients like FAs and glutamine.^218,219^ Secondly, inflammatory adipokines secreted by WAT, such as IL-6, leptin, and adiponectin, can influence STAT3, AKT, JNK, and MEK/ERK signal pathways in cancer cells directly, consequently upregulating the expression of proliferation and invasion-related genes.^220–222^ Finally, adipocytes are also involved in building a beneficial microenvironment for cancer cells. For example, adipocytes can decrease the natural killer cell toxicity, activate the inflammatory phenotype of macrophages, and promote fibrosis and vascularization in tumors.^223–226^ But compared to the non-thermal adipocytes, the pathophysiological mechanisms of brown or beige adipocytes that regulate malignant biological properties remain elusive in multiple types of cancer (Fig. 3).

### Cancer cachexia

Cancer-associated cachexia is a multifactorial syndrome characterized by weakness, loss of fat, and muscle wasting, which is driven by a variable combination of reduced food intake and metabolic changes, such as elevated energy expenditure, excess catabolism, and systemic inflammation. Moreover, cancer cachexia has commonly been considered the main inducement of complications in patients with malignancy, leading to reduced quality of life and poor outcomes.^227–230^ Over the years, plenty of evidence has shown that thermogenic fat contributes to cancer cachexia owing to its key role in heat production and energy balance.^231,232^ Brown or beige fat is an active tissue associated with hypermetabolism because of the high presence of UCP1, which leads to thermogenesis and energetic inefficiency. Thus, a switch from white to thermogenic fat contributes to cancer cachexia by increasing systemic energy expenditure. Notably, the WAT browning generally takes place during the initial steps of cancer cachexia, preceding the loss of muscle.^233–235^ During cachexia progression, the process of WAT browning can be triggered by pro-inflammatory mediators including IL-6 and the lipid-mobilizing factor zinc-α_2_-glycoprotein (ZAG),^219,236,237^ or by tumor-derived compounds like parathyroid-hormone-related protein (PTHRP),^238–240^ miR‐146‐5p,^241^ leukemia inhibitory factor (LIF),^242^ growth differentiation factor 15 (GDF15),^243^ or by sympathetic secretion, such as catecholamine.^244^ According to the fact that WAT browning usually occurs in the early stage of cancer cachexia, the biomarkers involved in browning are capable of becoming novel biochemical indicators for prognosis or potential targets for treatment.

### The adipose tissue microenvironment in cancer development and progression

The adipose tissue microenvironment (ATME) consists of multiple cell types, such as adipocytes, stromal cells, immune cells, vascular endothelial cells, and fibroblasts.^245^ Importantly, there’s plenty of evidence that ATME is associated with a variety of metabolic diseases, including malignancy.^246,247^ Based on currently available evidence, the ATME promotes tumor initiation and progression in multiple ways. First, adipocytes can secrete nutrients and adipokines into the microenvironment, contributing to cancer cell proliferation and invasion. Give an illustrative example, leptin released into ATME by white adipocytes can blind to the leptin receptor which is highly abundant in many tumors, then synergize with plenty of different oncogenes, cytokines, and growth factors by impacting the JAK-2/STAT, PI3K/AKT-1, and MAPK/ ERK1/2 signaling pathways.^248,249^ Second, it has been proved that adipose tissue accelerates cancer development and enhances tumor resistance to chemotherapy or immunotherapy through impinge on immune cells in the microenvironment. For instance, adipocytes release cellular contents into the microenvironment through pyroptosis or necrosis in response to external clues like hypoxia or low pO_2_ and mechanical stress, then triggering the accumulation of phagocytic macrophages consequently.^250,251^ Of note, their accumulation is frequently correlated to poor outcomes and therapeutic resistance in cancer patients.^252–254^ In addition, adipocytes are capable of recruiting other inflammatory cells by secreting chemokines into ATME, such as monocyte chemoattractant protein 1 (MCP1) and TNF.^255^ Next, infiltrating inflammatory cells like mast cells produce multiple proteases such as cathepsin S, leading to cancer progression and chemotherapy resistance. Besides, the elastase of neutrophils causes insulin resistance and elevated levels of free insulin by cleaving insulin receptor substrate 1 (IRS1), contributing to enhanced phosphoinositide 3-kinase (PI3K) signaling within tumor cells.^256–258^ Third, adipocytes can modulate endothelial cells and promote tumor vascularization to accelerate cancer progression. On the one hand, adipocytes in ATME strongly support tumor growth and enhanced angiogenesis by releasing particular molecules directly in hepatocellular carcinoma. On the other hand, adipocytes recruit and activate macrophages through a CCL2/IL-1β/CXCL12 signaling pathway, and activated macrophages in turn promote stromal vascularization and angiogenesis.^259,260^ Notably, the survival time of patients with breast cancer who are treated with anti-vascular endothelial growth factor (VEGF) is not extended. Potential mechanisms may include the upregulation of IL-6 and/or FGF-2.^261^ In addition, vascular inflammation caused by adipokines like leptin leads to elevated permeability, which is indispensable for cancer metastasis.^262,263^ Finally, adipocytes can affect the biological behavior of carcinoma by modifying the extracellular matrix (ECM) structure which is characterized by a high degree of fibrosis in ATME.^264^ Typically, the researchers observed an abundant fibrotic response in tumor areas that were enriched in adipocytes or located adjacent to adipose tissue. Moreover, the interaction between pancreatic stellate cells and adipocytes promotes matrix remodeling and impairs vascular perfusion, leading to tumor growth and ineffective chemotherapy.^265^ Given that, the multiple roles of white fat microenvironment have been described by many investigations during tumor initiation and progression. Nevertheless, how the thermogenic adipocytes in ATME affect tumor development and metastasis is mysterious. To make it clear, we may need to identify subpopulations of adipocytes in tumor-associated ATME by single-nucleus sequencing (sNuc-seq) or single-cell RNA sequencing (scRNA-seq) technique.

### The roles of thermogenic adipocytes differ in metabolism-related cancers

The interaction (except cachexia) between thermogenic adipose tissue and cancer cells differs in tumor types. Nowadays, our understanding of it is limited but promising, which is expected to provide a novel strategy for diagnosis, therapy, or prognosis.

#### Liver cancer

Hepatic carcinoma is the fourth leading cause of cancer-related mortality worldwide, which generally arises in a background of hepatitis and cirrhosis. Primary liver cancer commonly consists of hepatocellular carcinoma (HCC), intrahepatic cholangiocarcinoma (iCCA), and other rare tumors like hepatoblastoma and fibrolamellar carcinoma. On the basis of recent epidemic data, HCC alone accounts for 80–90% of primary liver cancers, and iCCA accounts for 10–15%.^266–268^ It is generally recognized that chronic inflammation is the most important contributor to the incidence and development of primary liver cancer. These chronic inflammation originate from hepatitis B or C virus (HBV or HCV) infections, metabolic disorders (including excess body weight, diabetes, impaired glucose tolerance, metabolic syndrome, alcoholic steatohepatitis (ASH), and nonalcoholic fatty liver disease (NAFLD), smoking, chronic toxin exposure, and parasite infection, and the majority of them are potentially modifiable risk factors.^269,270^ Notably, although vaccination programs and antiviral therapies have led to a decrease in HCC incidence, NAFLD has become the fastest-growing cause of HCC owing to the increasing prevalence of obesity.^271^ NAFLD is diagnosed using either imaging or a liver biopsy assessment with the presence of steatosis in more than 5% of hepatocytes in individuals, who consume little or no alcohol (<30 g per day for men and <20 g per day for women)and without metabolic risk factors (for example, obesity and type 2 diabetes) or other chronic liver diseases(for example, Wilson’s disease, congenital or acquired lipodystrophy).^272,273^ In general, NAFLD encompasses a disease continuum from nonalcoholic fatty liver (NAFL, the non-progressive subtype of NAFLD) to nonalcoholic steatohepatitis (NASH, the progressive subtype of NAFLD), which is characterized by necroinflammation and faster fibrosis progression than NAFL. Recently, there are some great reviews summarizing the pathogenetic mechanisms of the transition from NAFL to NASH and even HCC.^274–276^ Thus, we put more emphasis on the key roles of thermogenic adipocytes in the dynamic transformation process from NAFLD to HCC. According to recent investigations, thermogenic fat is induced to increase the energy expenditure of the whole body, and then alleviate obesity and hepatic steatosis simultaneously.^277–279^ Give some examples, exercise training is the most effective strategy to prevent obesity and NAFLD, partially because β-aminoisobutyric acid secreted from myocytes promotes a switch from WAT to thermogenic phenotype and enhances fatty acid β-oxidation in hepatocytes both through the PPARα signaling pathway, and perhaps IL-6-induced upregulated thermogenesis also play a part in alleviating NAFLD.^280,281^ In addition to aerobics, substantial strong evidence proved that some natural plant extracts could attenuate NAFLD by enhancing adipocytes browning and energy metabolism, such as the platycodon grandiflorus root (a Korean medicinal food, increases the expression of thermogenic genes like SIRT1, PPARα, PGC1A, and UCP1, which accompanied changes in fatty acid oxidation and energy expenditure),^282^ pomegranate seed oil (increases the levels of thermogenic genes and hepatic HO-1, along with decreased inflammatory adipokines and hepatic fibrosis),^283^ the Diospyros kaki fruit and Citrus unshiu mixture (activates fatty acid β-oxidation and thermogenesis, and inhibits lipogenesis and cholesterol synthesis via suppression of sterol regulatory element-binding protein 1 (SREBP-1) and SREBP-2 and its target genes),^284^ palmitoleic acid (increase lipid metabolism in adipocytes),^285^ cyanidin-3-O-β-glucoside (the most abundant monomer of anthocyanins, reduces adipokines secretion and lipid accumulation in HepG2 cells),^286^ the ARPS (polysaccharides from Anoectochilus roxburghii (Wall.) Lindl, promotes fat thermogenesis via the AMPK/SIRT1/PGC1A signaling pathway).^287^ Consistent with the above findings, activating thermogenesis in the protein level like the voltage-dependent anion channel 1 (VDAC1)-based peptide R-Tf-D-LP4 (a mitochondrial protein with multiple functions, like regulating cellular metabolism and energy expenditure) or disrupting the thermogenic suppressor gene nuclear receptor-interacting protein 1 (NRIP1, suppress glucose transport, fatty acid oxidation, mitochondrial respiration, UCP1 expression) by CRISPR enhances lipid metabolism in the liver, offering a promising therapeutic approach for liver steatosis.^288,289^ Conversely, the impairment of thermogenesis leads to metabolic disorders and liver steatosis.^290–292^ Of note, UCP1 in brown or beige adipocytes can be leveraged to antagonize inflammation of NAFLD. Mechanically, increased succinate in liver tissue drives inflammation through blinding to succinate receptor 1 (SUCNR1) in liver-resident stellate cell and macrophage populations and activated thermogenic adipocytes in mice protect against SUCNR1-dependent inflammatory infiltration in the liver.^293^ In conclusion, thermogenic adipocytes prevent the progression of NAFLD and thereby reduce the occurrence of HCC through increasing energy expenditure and suppressing liver inflammation. By contrast, the crosstalk between thermal adipocytes and hepatic cancer cells is barely revealed. Just one previous research found that the thermogenesis signaling pathway was upregulated in HCC patients without fibrosis by functional enrichment analysis, which might predict survival in HCC patients.^294^ Thus, comprehensive investigations on the interaction between thermal adipocytes and liver cancer cells are urgent and meaningful.

#### Renal cell carcinoma

Renal cell carcinoma (RCC) denotes cancer that originated from the renal epithelium and accounts for more than 90% of cancers in the kidney. Epidemiologically, represents the sixth most frequently diagnosed cancer in men and the tenth in women, respectively, and its incidence rates have been increasing. Based on histological and cytogenetic signatures, RCC is divided into several subtypes. Approximately 80% of RCC individuals are diagnosed with clear cell RCC (ccRCC), and up to a third of cases will present with or develop metastases.^295–297^ Of note, ccRCC can invade into surrounding perinephric adipose tissue (PAT), which distributes between the renal capsule and renal fascia, and this process is associated with some adverse perioperative outcomes and poor prognosis.^298–300^ Given that cancers are capable of reprogramming noncancerous neighboring cells to fuel tumor growth via providing additional nutrients, the potential protumorigenic relationship between ccRCC cells and PAT has been gradually studied in recent years. For example, recent research has shown that melatonin promotes tumor slimming and suppress tumor progression by activating transcriptional coactivator PGC1A and lipid browning programs.^301^ In agreement with this finding, PRDM16 is epigenetically silenced in RCC, and restoration of PRDM16 represses tumor growth. Specifically, researchers used RNA-Seq analysis to find that PRDM16 disrupts the transcriptome of cancer cells like semaphorin 5B (SEMA5B), which is a hypoxia-inducible factor (HIF) target gene highly expressed in RCC that promotes in vivo tumor growth.^302^ However, a new study has drawn the opposite conclusion that ccRCC promotes adipocyte browning to enhance tumor growth. More precisely, ccRCC cells activate PAT browning through the secretion of PTHRP, then the thermogenic adipocytes increase lactate secretion and promote ccRCC cell proliferation. Notably, tyrosine kinase inhibitors (TKI) often used to treat ccRCC, such as sunitinib, have been shown to activate adipocytes browning, and the combination therapy of TKI plus browning inhibitor present a more-complete suppression of ccRCC.^303^ The mentioned above result seems to be controversial for now, but it will be explained clearly after systematically analyzing transcriptomics and metabolomics of cancer cell-reprogrammed adipocytes and comprehensively clarifying how browning of adipose tissue impacts other members in the tumor microenvironment (TME), such as immune cells.

#### Pancreatic cancer

Pancreatic cancer (PC) is one of the most serious diseases which has a poor outcome and its five-year survival rate remains less than 10% in the U.S. and 7.2% in China, which is often due to a lack of early detection and effective treatment. Besides, extensive research has shown that PC also ranks 4th and 6th the cancer-associated deaths in the U.S. and China, respectively. What’s more, PC has an increasing incidence of 13 per 100,000 people per year, and it is projected to become the second leading cause of cancer-related deaths in the U.S. by 2030.^2,6,304,305^ Multiple evidence has shown that obesity and T2DM are modifiable risk factors associated with the development of PC, which implies that the metabolic abnormity of the whole-body fat contributes to tumor progression.^265,306,307^ Previously, researchers found a phenotypic switch from WAT to brown fat in Kras-pancreatic cancer mice, which was associated with high-expressed UCP1 caused by chronic inflammation and IL-6.^234^ Consistent with this finding, a recent study described 3 phases of metabolic and soft-tissue changes before PC diagnosis, and the reduction of subcutaneous adipose tissue (SAT) was observed during phases 2 and 3 (start 18 months before PC identification). In addition, SAT wasting was likely related to the browning of adipocytes, because overexpression of UCP1 in SAT exposed to PC exosomes was tested in mice and patients with PC.^308^ Mechanically, the browning of SAT may be induced by exosomal adrenomedullin shed from cancer cells via activating the p38 and the extracellular signal-regulated kinase (ERK)1/2 and the mitogen-activated protein kinases (MAPKs) signaling axis.^309^ Of note, it is commonly believed that thermogenic fat can accelerate the intake of glucose and improve insulin resistance in obese and T2DM. However, hyperglycemia is detected in quite a few PC patients before or after the diagnosis of PC, although the browning of SAT is considered a phenomenon of decreasing blood glucose.^310–312^ Revealing the underlying mechanism of this seemly controversial phenomenon promisingly provides a novel view for new-onset diabetes in patients with PC. In addition to SAT, pancreatic fat accumulation is linked to chronic pancreatitis, pancreatic neoplasms, disturbed glucose metabolism, and impaired insulin secretion. Given that some cancers have been observed to promote browning of adjacent WAT, whether the browning of intrapancreatic adipose tissue occurs and the crosstalk between brown pancreatic fat and tumor required further research.^215,313,314^

#### Breast cancer

Breast cancer (BC) is the most common cancer diagnosed in many countries including the US (excluding skin cancers) and China, which is the second leading cause of cancer death among women after lung cancer and remains the primary tumor-associated cause of disease burden for women. In addition, it is important to notice that BC is also the most commonly diagnosed cancer type in young adults aged 30 to 39 years.^315–318^ In line with PC, overweight and obesity are highly related to the prevalence of BC in postmenopausal females. Moreover, the breast adipose tissue is impacted by surrounding cancer cells, and vice-versa modifies the TME in favor of cancer via browning of WAT.^319^ For example, a recent study demonstrated that markers for BAT and beige adipocytes were highly expressed in BC xenografts, implicating that thermal characteristics could play a vital role in BC progression.^320^ In agreement with this finding, newly published articles have suggested that BC mammospheres can secrete adrenomedullin to induce the browning of adjacent adipocytes and lipolysis. Furthermore, activated thermogenic adipocytes in the breast can modulate the behavior of mammary epithelial cells and promote tumor progression in both tumor and non-tumor mice.^321,322^ In addition, a large cohort clinical trial showed that chemotherapy harmed the activity of BAT, which maybe explain why weight gain during chemotherapy.^323^ In conclusion, comprehensively understanding the interaction between BC cells and adjacent adipose tissue will path a novel way to combat breast tumor progression.

#### Gastric cancer

Tumors derived from the stomach are a global health problem, with more than 1 million people newly diagnosed with gastric cancer (GC) worldwide each year. Although chemotherapy, radiotherapy, surgery, and immunotherapy all have proven efficacy in GC, it does represent the third most common cancer-related death worldwide and is responsible for >700,000 deaths annually.^324–326^ There are various factors contributing to tumorigenesis of the stomach, including Helicobacter pylori infection, lifestyle factors, and genetic risk factors.^327^ Recently, substantial preclinical experiments and clinical trials have demonstrated that adipose tissue in a particular position (such as SAT) is associated with the prognosis of GC patients.^328–332^ However, the function of WAT browning in GC was reported by merely several articles. For instance, researchers found that exosomes released from GC cells could deliver circular RNAs-133 into preadipocytes, promoting the differentiation of preadipocytes into mature adipocytes with thermogenic phenotype via stimulating PRDM16 and suppressing miR-133.^333^ Similarly, a new study has suggested that exosomal miR-155 from GC suppresses adipogenesis and enhances brown adipocytes differentiation in adipose mesenchymal stem cells via CCAAT/enhancer-binding protein (C/EBP) β, which causes cancer-associated cachexia.^334^ In general, more research is needed for understanding the impact of browning on other GC phenotypes.

#### Colorectal cancer

Colorectal cancer (CRC) is the main contributor to global cancer mortality, accounting for roughly 1.9 million new cases and 0.9 million deaths per year worldwide. Optimistically, the incidence and mortality are gradually stable and even slightly declined in developed countries owing to nationwide screening programs and increased uptake of colonoscopy in general. Nevertheless, new cases of early-onset CRC (generally defined as CRC diagnosed before the age of 50 years) have recently been increasing globally, which exhibits different clinical manifestations, pathological characteristics, and molecular features compared to later-onset CRC patients. As with most cancers, body fat and obesity are modifiable risk factors increasing CRC incidence.^335–338^ Although the biofunction of thermogenic adipocytes is less well understood, it has engaged researchers’ interest recently. Give an example, they find that intestinal disease tolerance is preferentially established in thermoneutral mice, protecting them from injury-induced colitis and inflammation-induced colon cancer. Besides, the underlying mechanism is mediated by an unexpected crosstalk between thermogenic adipocytes and intestinal epithelial cells.^339^ In addition, more direct evidence has shown that the expression of UCP1 is significantly associated with better overall survival of CRC in a cohort study.^340^ Furthermore, a signature consisting of six biomarkers, including UCP1, signal transducer and activator of transcription 1 (STAT1), p-cofilin, LIM domain kinase 2 (LIMK2), the Forkhead transcription factor family member 3 (FOXP3), and inducible co-stimulator (ICOS), was identified as the best combination in terms of prognostic power.^341–343^ However, it is temporally unclear how thermogenic signaling improves the overall survival of CRC patients, so further study is necessary.

The clue of thermogenic adipose tissue is indirect and scattered in the rest of the cancers, thus which will not be discussed here. Collectively, cancer cachexia is the most common and mechanically clear impact caused by WAT browning. In contrast, the local inter-communication between thermogenic adipocytes and other cells in TME-like immune cells is unrevealed. Therefore, future research on this topic should be encouraged.

### Clinical applications

Findings over the past two decades have comprehensively described the multiple functions of thermogenic adipocytes in mice and humans. Significantly, their abnormal activity is closely related to cancer and other metabolic diseases. Accordingly, investigators have been actively searching for effective tools and pharmacologic agents to identify and manipulate activated thermogenic fat, respectively, holding promise for combating malignancy and metabolic disorders clinically.

### Identification of thermogenic fat

Different from WAT, thermogenic fat has distinctive features of ontogeny, bioenergetics, and physiological functions. These characteristics provide an opportunity to differentiate thermogenic fat from WAT by using imaging tools.^344^ Here, several common imaging methods for the assessment of thermogenic fat will be discussed in detail. Additionally, some novel molecular imaging modalities will be enumerated in Table 1.

**Table. 1:** Main imaging methods currently used to detect thermogenic fat

Imaging modality	Imaging mechanism	Imaging subject	Advantages	Disadvantages
PET				
^ 18^F-FDG	Glucose metabolism	Rodent/human	Widely used; shows BAT activation; short acquisition time	Ionizing radiation; high cost easily affected by imaging conditions
^ 18^F-THA	Fatty acid metabolism	Rat/human	BAT activation-dependent	Ionizing radiation; limited availability
^ 18^F-FBnTP	Mitochondria membrane potential	Rat	High accumulation in inactive BAT; highly sensitive and rapidly responsive to BAT	Decreased signal after BAT activation
^ 18^F-F-DPA	TSPO ligand	Mouse	highly specific and practically mature	Ionizing radiation; high cost
^ 15^O–O_2_	Oxygen consumption	Human	Direct measurement of oxygen consumption; the uptake increased with BAT activation	Short half-time; limited availability
^ 11^C-acetate	Oxidative activity	Rat/human	Activation-dependent uptake	Short half-time
MR				
Chemical-shift water-fat MRI	Fat-water content	Rodent/human	Widely used; no ionizing radiation	Limited in BAT/WAT mixture differentiation
T2* mapping	Mitochondria and oxy/deoxyhemoglobin	Mouse/human	Widely used; no ionizing radiation	Limited in BAT/WAT mixture differentiation
BOLD	Oxygen consumption and blood flow	Rodent/human	Dynamic detection of BAT;	Limited in susceptibility and breathing artifacts
Hyperpolarized Perfusion of MRI	hyperpolarized ^13^C or ^129^Xe probes	Mouse	Dynamic detection of BAT	Limited in hyperpolarization technique
Others				
SPECT	Mitochondrial density; sympathetic	Rodent/human	Detect BAT density and mitochondrial activity	Ionizing radiation; low-resolution innervation
CEUS	Blood flow	Mouse/human	No ionizing radiation; low cost; dynamic imaging	Operator-dependent; limited penetration depth
IRT	Skin temperature	Human	No ionizing radiation;	Limited penetration depth low cost; convenient; readily repeatable
NIRS	Oxygen content	Human	Simply operate; low cost	Limited penetration depth

*PET* positron emission tomography, ^*18*^*F-FDG* ^18^F-fluorodeoxyglucose, ^*18*^*F-THA 14-(R,S)* ^18^F-flfluoro-6-thiaheptadecanoic acid, ^*18*^*F-FBnTP* ^18^F-flfluorobenzyltriphenyl phosphonium, ^*18*^*F-F-DPA* N,N-diethyl-2-(2-(4-(^18^F-flfluoro)phenyl)-5,7-dimethylpyrazolo[1,5-a]pyrimidin-3-yl)acetamide, *TSPO* translocator protein MRI magnetic resonance imaging, *BOLD* blood-oxygen-level dependent, *NIRF*I near-infrared fluorescence imaging, *SPECT* single-photon emission imaging computerized tomography, *CEUS* contrast-enhanced ultrasound, *IRT* infrared thermography, *NIRS* near-infrared spectroscopy.

#### PET

PET is the most frequently used imaging method for the assessment of thermogenic adipose tissue, and ^18^F- FDG-PET/CT is currently the most frequently used method for visualizing activated thermogenic fat in mammals and humans. ^18^F-FDG-PET/CT scans are commonly conducted for cancer diagnosis, staging, and surveillance. Clinicians can identify suspected malignancies and metastases by measuring the uptake of radiolabelled glucose.^345^ Since thermogenic adipocytes are good at intaking glucose in the same way, reports firstly described increased uptake of the glucose analog in supraclavicular fat two decades ago, suggesting the presence of metabolically active BAT in adult humans.^346^ After that, plenty of research sprung up and proved the prevalence of active thermogenic adipose tissue in humans. Moreover, investigators developed Brown Adipose Reporting Criteria in Imaging Studies (BARCIST 1.0) criteria to produce the diagnosis based on scoring thermogenic fat.^67,68,135,347,348^ In recent years, ^18^F-FDG-PET/CT is used to assess the distribution and activity of adipose tissue, rather than diagnosing and staging cancers.^349^ A series of clinical trials have demonstrated that BAT activity on ^18^F-FDG-PET/CT is prevalent and it is negatively associated with lymph nodes and distant metastasis.^350,351^ In contrast, a new clinical study consisting of multiple cancers suggested that elevated thermogenic fat volume is linked with lower cancer-associated death.^352^ Furthermore, another study suggested that patients with active cancer have more BAT compared to patients with successfully treated cancer.^353^ In conclusion, ^18^F-FDG-PET/CT of thermogenic fat could serve as a novel measurement for the outcome of cancer patients, but more large clinical trials are needed.

#### MRI

Although ^18^F-FDG-PET/CT remains the gold standard for the detection and quantification of thermogenic fat in humans, other novel imaging techniques have sprung up like magnetic resonance imaging (MRI). Compared with PET, MRI has better spatial resolution at a lower cost and is much safer as they do not involve the injection of radioactive tracers.^354,355^ There are already some comprehensive and updated reviews of MRI mechanisms and applications for thermogenic adipose tissue, so we mainly focus on its role in tumors.^356–358^ In *KRAS*^G12D^ *P53*^R172H^ Pdx‐Cre^+/+^ (KPC) mouse, tumor progression induced loss of fat thermogenesis, which was detected by MRI.^359^ Consistently, the water-fat separated MRI could identify and quantify the BAT in C57BL/6 mice that were inoculated orthotopically with Pan02 tumor cells.^360^ Furthermore, dynamic wasting of BAT can be captured by a dedicated high-Fifield (7 Tesla) Bruker 7T ClinScan MRI combined with an infrared camera in the ovarian tumor mouse model.^361^ Unfortunately, the information about MRI identifying thermogenic fat in clinical patients with malignancy is little.

#### Imaging of thermogenic fat using other technologies

Apart from PET and MRI, plenty of other modalities have been developed to identify thermogenic adipose tissue, such as single-photon emission computerized tomography (SPECT), near-infrared fluorescence imaging (NIRFI), contrast-enhanced ultrasound (CEUS), near-infrared spectroscopy (NIRS), infrared thermography (IRT).^344,355^ These advanced imaging techniques have their advantages and disadvantages, respectively, and further study is necessary for validating their practical applications.

### Therapeutic strategies of thermogenic fat

Given the role of thermal fat in nutrient metabolism and energy expenditure as well as its impact on other tissues, activating thermogenic adipose tissue provides a promising therapeutic strategy for curbing obesity, T2DM, and other metabolic diseases. Although the overwhelming benefits have been demonstrated in some metabolic diseases, the adverse side effects of browning should not be ignored, like cachexia and cardiovascular events in hypermetabolic conditions. In turn, blocking WAT browning might be exploited for clinical benefit in hypermetabolic patients. Collectively, we have to figure out how we can preserve the therapeutic effects by manipulating thermogenesis, while also eliminating many of the unexpected side effects.^362–364^ Here, we summarize current clinical trials of targeting thermogenic adipose tissue pharmacologically for metabolic diseases in Table 2. In addition, we also cite some evidence to show the therapeutic potential of brown and beige fat in humans with malignancy.

**Table. 2:** Current clinical trials targeting activation or inhibition of thermogenic fat in humans

Type of therapy	Pharmacologic agent	Study participants	Phase	Status	Trials ID
Pro-browning					
β3-AR agonist	Mirabegron	Healthy volunteers	I	Recruiting	NCT01950520
	Mirabegron	Healthy volunteers	Not applicable	Completed	NCT02811289
	Mirabegron	Healthy volunteers	I	Recruiting	NCT03049462
	Mirabegron	Healthy volunteers	I/II	Not yet recruiting	NCT04766021
	Mirabegron	Healthy volunteers	Not applicable	Recruiting	NCT04823442
	Mirabegron	Healthy volunteers	II	Completed	NCT01783470
	Mirabegron	People with obesity	I	Completed	NCT02919176
	Ephedrine	Healthy volunteers	Not applicable	Completed	NCT01015794
	Ephedrine and caffeine	People with obesity	III	Completed	NCT02048215
	Ephedrine and pioglitazone	Healthy volunteers	Not applicable	Recruiting	NCT02236962
Thyroid hormones	Levothyroxine	Patients with thyroidectomy	Not applicable	Completed	NCT02499471
	Levothyroxine	Healthy volunteers	IV	Recruiting	NCT01379170
	Liothyronine	Patients with insulin receptor mutation	II	Completed	NCT02457897
Capsaicinoids	Capsinoids	Healthy volunteers	Not applicable	Not yet recruiting	NCT02964442
	Capsinoids	People with obesity	Not applicable	Completed	NCT03110809
	Capsinoids	Healthy volunteers	Not applicable	Completed	NCT01961674
GLP1	Liraglutide	People with obesity	III	Not yet recruiting	NCT02718950
	Liraglutide	People with T2DM	IV	Terminated	NCT01638260
	Liraglutide	People with obesity	III	Not yet recruiting	NCT02718950
	Bydureon	Healthy volunteers	IV	Recruiting	NCT03002675
	Bydureon	People with obesity	III	Completed	NCT00856609
	Sitagliptin	People with impaired glucose tolerance	IV	Completed	NCT02294084
Other agents	Bromocriptine	Healthy volunteers	Not applicable	Completed	NCT02428933
	Nicotinic acid	People with T2DM or not	Not applicable	Recruiting	NCT05092945
	Creatine	People with a vegetarian diet	Not applicable	Completed	NCT04086381
	L-arginine	People with impaired glucose tolerance	III	Recruiting	NCT02291458
	Adenosine	Healthy volunteers	Not applicable	Completed	NCT03327168
	Fluvastatin	Healthy volunteers	IV	Completed	NCT03189511
	Niagen	Healthy volunteers	Not applicable	Completed	NCT02835664
Anti-browning					
Anti-thyroid drug	CMZ or TMZ	Patients with Graves’ disease	Not applicable	Not yet recruiting	NCT03064542
Glucocorticoids β3-AR blocker	Prednisone	Healthy volunteers	IV	Completed	NCT03269747
	Propranolol	Healthy volunteers	Not applicable	Completed	NCT01791114
	Propranolol	People with hyperthyroidism	IV	Completed	NCT03379181
	Propranolol	Healthy volunteers	II	Recruiting	NCT01950520

*β3-AR* β3-adrenergic receptor, *GLP1* glucagon-like peptide 1, *T2DM* type 2 diabetes mellitus, *CMZ* carbimazole, *TMZ* thiamazole, *Niagen* nicotinamide riboside.

#### Targeting activation of thermogenic adipose tissue

Activating the browning of adipose tissue is emerging as an interesting and promising method to curb metabolic disorders like obesity and T2DM because of its unique capacity to upregulate non-shivering thermogenesis.^365^ The most physiological external clues stimulating the thermogenesis of adipose tissue include cold exposure, diet, and exercise.^366^ Both the short-term cold exposure (10 °C, 4 h) and chronic cold exposure (6 °C, 10 days) induce WAT browning and UCP1-mediated thermogenesis-dependent glucose utilization.^367,368^ Further, intermittent cold exposure increases BAT thermogenesis and improves insulin sensitivity in humans consequently.^369^ In addition, exercise enhances glucose homeostasis and protects against metabolic diseases such as obesity and diabetes.^370^ Recently, investigators have demonstrated that exercise induces the phenotypic conversion of white adipocytes to thermogenic adipocytes through a range of mechanisms.^371^ During exercise, increased plasma epinephrine stimulates lipolysis through acting cAMP, PKA, and phosphorylates adipose triglyceride lipase (ATGL). Moreover, it has been demonstrated that exercise can stimulate the browning of WAT and increase energy consumption by promoting the expression of hypothalamic brain-derived neurotrophic factor (BDNF).^372^ Of note, various food ingredients have been considered potent stimuli inducing a phenotypic switch from white to beige adipocytes and activating thermogenesis. For instance, capsaicin and its analog capsinoids mimic the effects of cold exposure to decrease body fatness through the activation and recruitment of BAT through activating transient receptor potential (TRP) channels.^373^ Interestingly, intermittent fasting stimulates the thermogenic activity of BAT, which contributes to preventing obesity via altering the composition of the microbiota in mice.^374^ Besides, a variety of phytochemicals, including phenylpropanoids (e.g., artepillin C, resveratrol), flavonoids (e.g., catechin, quercetin), terpenoids (e.g., ginsenoside, fucoxanthin), alkaloids (e.g., capsaicin, caffeine), glycosides (e.g., oleuropein), and phenolic acid (for example, gallic acid) have been shown in the literature to counteract weight gain via adipose thermogenesis.^155,375,376^ Compared with the above approaches for activating thermal fat function, pharmacological products have a more specific effect with minimized adverse consequences (Table 2). Remarkably, recent preclinical investigations have shown that successful BAT transplantation models display improvements in glucose metabolism and insulin sensitivity, as well as reductions in body mass and decreased adiposity in recipients. These beneficial effects are mediated by several different mechanisms, including endocrine effects via the release of batokines.^377^ With the view of clinical application, progenitors or stem cell therapy is recognized as a more feasible strategy than tissue transplantation. Give an illustrative example, human adipose-derived stem cells were differentiated into brown adipocytes with rosiglitazone and then injected into mice every other week over 10 weeks, then the models injected with the thermogenic cells showed a loss of body weight and enhanced glucose tolerance.^378^

Although driving thermogenesis in preclinical and clinical studies of metabolic diseases like obesity showed exciting results, its beneficial effects on cancer patients have not been reported yet. Regarding previous investigations, the abnormal accumulation of WAT promotes tumorigenesis, progression, invasion, and metastasis.^379^ Thus, locally activating WAT browning and lipolysis, such as in the fatty liver and pancreas, might suppress oncogenesis prophylactically or inhibit tumor growth by an unknown signaling way. All of these hypotheses require strong evidence.

#### Targeting inhibition of thermogenic adipose tissue

On present evidence, hypermetabolic conditions including burns and cancer in which browning is detrimental to patient outcome.^363^ Now, activating thermogenic fat accelerates cancer-associated cachexia and promotes tumor growth, which implies that blocking the browning of adipose tissue seems to be an advantageous treatment for individuals with malignancy. In the skin tumor mice model, investigators found that WAT browning was an early event in the pathophysiology of cancer cachexia, and treatment with the selective β3-AR antagonist or nonsteroidal anti-inflammatory drug sulindac could ameliorate cachexia owing to the reduction of browning in subcutaneous WAT.^234^ In agreement with that, using the neutral sphingomyelinase inhibitor GW4869 or Arctii Fructus (the extract of Arctium lappa) improves cancer-induced cachexia and decreased mortality by inhibiting browning.^380,381^ Of note, Metformin, the first-line drug treatment for hyperglycemia and insulin resistance, has been considered a novel anticancer agent unexpectedly. The primary reason is that Metformin can inhibit the electron transport chain (ETC) and ATP synthesis, and it also can regulate AMP-activated protein kinase (AMPK) and mTORC1 in multiple ways.^382,383^ Furthermore, recent research demonstrated that treatment with Metformin could improve the tumor-induced wasting state and minimize cachexia in tumor-bearing rats, probably it prevented the pathological browning of WAT.^384,385^ So from the point of translational medicine, classical anticancer treatment combined with inhibiting fat browning might provide a promising option for patients.

## Conclusions and perspective

Thermogenic adipocytes including brown and beige adipocytes have garnered considerable attention recently, mainly because both of them have similar impacts on thermoregulation and nutrient utilization. Although brown and beige adipocytes come from distinct origins during embryonic development and locate in different positions anatomically, they have multilocular lipid droplets, abundant mitochondria, and highly expressed UCP1. Of note, beige fat cells are reproducible in adults. More precisely, the emergence of beige adipocytes is an inducible process stimulated by external clues such as cold, exercise, and fasting. Therefore, manipulating the thermogenic program provide a promising therapeutic strategy for combating obesity, T2DM, cancer, and other diseases. Although multiple therapeutic strategies activating thermogenesis have been shown to work well in animal models, the majority of them do not apply to humans. Probably the heterogeneity in thermogenic adipose tissue between the two species make the translational application from mice to humans harder. Moreover, it should not be ignored that targeting thermogenic fat can give rise to adverse effects in vivo, such as cachexia, heart diseases, and other hypermetabolic disorders. Collectively, investigators and clinicians must consider it prudent how we can preserve the beneficial metabolic effects of browning and meanwhile eliminate many of the unexpected side effects.

In contrast to obesity or diabetes, the influence of thermogenic adipocytes is not one-sided anymore but double-edged during the initiation and progression of the tumor. Firstly, activated thermogenic adipocytes can prevent obesity, T2DM, and other metabolism-related diseases like NAFLD through increasing energy expenditure, enhancing glucose tolerance, or alleviating inflammation, and the majority of these metabolic diseases are generally considered high-risk factors for multiple tumors.^366,386^ However, the browning of adipocytes seems to support progression after tumor formation. Specifically, enhanced thermogenesis in SAT speeds up fat mass loss and cancer cachexia, thereby contributing to the poor outcome of cancer patients.^387,388^ In addition, the local activation of thermogenic adipocytes adjacent to the tumor can promote the proliferation and invasion of cancer cells by providing fuel sources like lactate. Ultimately, malignant cells are resistant to chemotherapy partly because chemotherapeutic drugs stimulate reinstallation of the thermogenic phenotype in WAT.^303^ Accordingly, the affection for manipulating thermogenic adipocytes is seemly quite different from preventing the onset of cancer to slowing tumor progression. Although targeting thermogenic adipocytes is not a reliable treatment for cancer patients based on current research, it has already presented its therapeutic potential in other metabolic conditions.^152,389–392^ Thus, the therapeutic strategy of regulating thermogenesis is an alternative option for metabolism-related cancer patients, if continue to conduct more research. Referring to the current status of investigating thermogenic fat, there are several aspects worth studying further in oncology.^393–395^ First, for example, it would be interesting to identify subpopulations of adipocytes or other cells in ATME in the different tumor contexts, to determine the origin of adipocytes, as well as their fate and phenotype (whether these cells form non-thermal or thermal fat, and the type of component they secrete). Besides, from the perspective of translational medicine, a novel detective technology is required for precisely distinguishing thermogenic fat from tumors and continuously evaluating the capacity of thermogenesis. Moreover, it is also necessary to explore how to achieve safe and effective beige fat activation in tumors locally, because inducing thermogenesis through traditional ways like cold stimuli or beta-adrenergic signaling possibly results in potential cardiovascular hazards.^161^

In conclusion, recent investigations provide new insights into the biology and pathology of thermogenic fat and preliminarily reveal its connection to metabolic diseases and malignancy, thereby suggesting that modulating the activity of thermogenic adipocytes holds promise for combating obesity, T2DM, cancer, and other metabolic diseases.
